# Minor Physical Anomalies in Bipolar Disorder—A Meta-Analysis

**DOI:** 10.3389/fpsyt.2021.598734

**Published:** 2021-06-16

**Authors:** Eszter Varga, András Hajnal, Alexandra Soós, Péter Hegyi, Dóra Kovács, Nelli Farkas, Júlia Szebényi, Alexandra Mikó, Tamás Tényi, Róbert Herold

**Affiliations:** ^1^Department of Pediatrics, Medical School, University of Pécs, Pécs, Hungary; ^2^Department of Psychiatry and Psychotherapy, Medical School, University of Pécs, Pécs, Hungary; ^3^Institute for Translational Medicine, Medical School, University of Pécs, Pécs, Hungary; ^4^Institute of Bioanalysis, Medical School, University of Pécs, Pécs, Hungary; ^5^Department of Dermatology, Venereology and Oncodermatology, Medical School, University of Pécs, Pécs, Hungary

**Keywords:** minor physical anomalies, bipolar disorder, Méhes Scale, Waldrop scale, neurodevelopment

## Abstract

**Introduction:** Minor physical anomalies (MPAs) may reflect basic neurobiological features underlying bipolar disorders (BPD), as they are sensitive physical indicators of morphogenetic failure of the brain. Despite several researches about the presence of MPAs in BPD, the results are still controversial.

**Objectives:** The aim of the present meta-analysis was to assess the standardized weighted mean effect sizes of MPAs in BPD and to examine if MPAs may be found predominantly in the head and/or facial regions in BPD patients compared to controls (HC).

**Methods:** Four studies, involving 155 patients with BPD, and 187 HC, were involved in the analysis after searching the literature. For the investigation of MPAs in the peripheral (MPA-P) and in the head and facial regions (MPA-CF), two studies involving 121 BPD patients, and 133 HC passed the inclusion criteria.

**Results:** The number of the MPAs in the BPD group was significantly higher compared to HC. Another important finding of the present study is that BPD patients' MPA-P scores do not significantly differ from those of the HC. In contrast, BPD patients' MPA-CF scores were found to be significantly higher compared to HC subjects. It is important to note that there was a low number of eligible publications included, which caused higher heterogeneity.

**Conclusions:** Low quality of evidence suggests that MPAs are more common in patients with BPD than in HC and the higher rate of MPAs is found predominantly in the head and facial regions.

## Introduction

BPD is diagnosed in more than 1% of the population irrespective of ethnicity, nationality, and social or economical status. It represents one of the most common reasons of disability among young individuals (1). Thus, understanding the pathophysiology and the etiology of the disease is important.

Recent literature suggests that BPD can be conceived as a neurodevelopmental disorder, at least in a subset of patients (1–4).

To explore the neurobiological background of BPD, it is important to find endophenotypic markers, besides finding genetic vulnerability. These markers are specific to the disease, reflect basic neurobiological features, and can be found during remission and also among unaffected relatives of patients with bipolar disorder (5). Among others, structural and functional neuroanatomical alterations, cognitive deficits, and developmental abnormalities, such as MPAs, were proposed as endophenotypes.

MPAs are non-significant failures of morphogenesis, which persist into adult life and are easy to examine visually. They develop during the first and early second trimesters of gestation (6–10). As both the central nervous system and the skin are developed from the ectodermal tissue, MPAs may be external markers of abnormal brain development and their presence is a sensitive physical indicator of embryonic development.

Earlier studies found that MPAs of the head and the mouth may have more relevance to the neurodevelopmental failure (4, 7, 9, 11–13). They also suggested that MPAs at different localizations represent different origins, such as familial or non-familial. Tikka et al. (14) discussed that MPAs of the craniofacial region (MPA-CF) were significantly more often found in the relatives of schizophrenia patients than in healthy controls. Aksoy-Poyraz et al. (15) found anomalies in the ear and the limb, greater head circumference, and intercanthal width, both in patients with schizophrenia and in their healthy siblings. These outcomes show that these anomalies represent familial predisposition for schizophrenia (15). Similar correlations of MPAs between schizophrenia patients and relatives were reported for the eyes, hands (16), and ears (16, 17). As far as we know, there are no such findings with BPD patients. During the last two decades, our research group has published important findings about the excess of MPAs in psychoses and Tourette syndrome (7, 18–20) and proposed using them for prognostic and diagnostic purposes (8).

In our earlier systematic review (21), we found that several studies show controversial results about MPAs in BPD, so meta-analysis could provide an objective average. Thus, the primary aim of this meta-analysis was to integrate findings of studies concerning the appearance of MPAs in patients with BPD. Based on former data, our first hypothesis was that MPAs may be more common in BPD than in normal subjects. As previous findings suggested, anomalies of the head and the mouth may have more relevance to the hypothetical neurodevelopmental failure (4, 7, 9, 11–13), and it is theorized that MPAs at different localizations may represent different origins, such as familial vs. non-familial, so our second hypothesis was that a higher rate of MPAs may also be found predominantly in the head and facial regions in BPD compared to normal subjects.

## Methods

### Literature Search, Study Selection, and Data Collection

Our meta-analysis followed the Preferred Reporting Items for Systematic Reviews and Meta-Analyses (PRISMA) guideline (22). We registered the study in the PROSPERO registry (registration number: 137481). The present meta-analysis was based on the patient, intervention, comparison, and outcome (PICO) format (P: adults with bipolar I and bipolar II disorders; I: MPAs; C: healthy controls; O: number of MPAs). We searched peer-reviewed databases including PubMed (*via* MEDLINE), Web of Science, Embase, and CENTRAL (up to April 2019) using the following search keys: (“minor physical anomalies” OR MPA) AND (“bipolar disorder” OR depression OR “affective disorders” OR “mood disorders” OR dysthymia). Reference lists of included studies and reviews were searched for additional studies. We selected studies that investigated MPAs in bipolar disorders, fulfilling DSM-III-R (23) or DSM-IV-TR (24), included healthy comparison groups, and used the Waldrop scale or Méhes Scale. All identified publications were reviewed, and data were extracted by two authors (H.A. and V.E.) independently. For MPA score calculations, means and standard deviations were extracted from the articles in patients with bipolar disorders and in the control groups as well. Consensus was reached to resolve any doubts.

### Assessment of Study Quality

The Newcastle–Ottawa Quality Assessment Scale (NOS) (25) risk-of-bias tool for case–control studies was used to assess the quality of included studies. It contains eight items, which are categorized into three dimensions, which are selection, comparability, and exposure. The adapted items of the NOS to this review, as well as the definitions of low-quality and high-quality items, are shown in Table 1. Details on methodological quality were identified during the data extraction process. Two authors (VE and HA) independently assessed the quality of the included studies (Table 2). A consensus method was used to resolve disagreement.

**Table 1:** The adapted items of the Newcastle–Ottawa Quality Assessment Scale (NOS) to this review.

	**Adapted Newcastle–Ottawa Scale items**	**High-quality items carrying a low risk of bias (green)**	**Low-quality items carrying a high (red) or an unknown (yellow) risk of bias**
Selection	Item 1: all patients diagnosed according to DSM criteria.	All patients with bipolar I and II fulfilled DSM criteria	Low: no
	Item 2: representativeness of the cases	All patients with bipolar disorder were included.	Low: any selection criteria were applied to the study population Unknown: no data on selection process.
	Item 3: selection of controls	Controls were selected from the same source population as the cases.	Low: controls were selected from a different source population as the cases. Unknown: no description.
	Item 4: definition of controls	Control with personal or family history of psychotic disorders, affective disorders, or other neuropsychiatric disease were excluded.	Low: definitions did not match the criteria listed in the high-quality column. Unknown: no definition provided.
Comparability	Item 5: study controls for sex	No significant difference was detected between patients with bipolar disorders and controls regarding sex.	Low: significant difference was detected between patients with bipolar disorders and controls regarding sex. Unknown: no comparison made by sex.
	Item 6: study control for age	No significant difference was detected between patients with bipolar disorders and controls regarding age.	Low: significant difference was detected between patients with bipolar disorders and controls regarding age. Unknown: no comparison made by age.
Outcome	Item 7: ascertainment of exposure	Examiner blinded to case/control status.	Low: unblinded examiners Unknown: no statement.
	Item 8: same method of ascertainment for cases and controls	Yes	Low: no

**Table 2:** Characteristics of the included studies.

**References**	**Cn**	**S.T**.	**Group**	**S.S**.	**Age**		**Gender (*****n*****)**		**Age at onset**		**Duration of illness**		**Scale**	**Total MPA**		**MPA-P**		**MPA-FC**	
					**Mean**	**SD**	**M**.	**Fm**.	**Mean**	**SD**	**Mean**	**SD**		**Mean**	**SD**	**Mean**	**SD**	**Mean**	**SD**
Berecz et al. (21, 26)	HU	Case–control	Bipolar I	30	52.30	10.00	–	–	52.30	10.00	28.80	8.30	Méhes	1.00	1.017	0.13	0.35	0.87	0.93
			Bipolar II	30	52.10	14.10	–	–	52.10	14.10	15.80	8.80		0.77	1.070	0.13	0.35	0.63	0.89
			Control	30	51.40	12.00	–	–	–	–				0.13	0.350	0.07	0.25	0.07	0.25
Akabaliev et al. (27)	BG	Case–control	Bipolar I	61	38.15	14.80	25	36	–	–	10.57	11.60	Modified Waldrop scale	4.85	1.840	1.32	1.00	3.51	1.79
			Control	103	39.65	10.68	49	54	–	–				3.07	1,830	0.49	0.85	2.15	1.59
Green et al. (28)	USA	Case–control	Bipolar	26	26.30	7.20	13	13	–	–	–	–	Modified Waldrop scale	1.23	1.030				
			Control	40	40.00	24.40	20	20	–	–				0.95	1.060				
Alexander et al. (29)	USA	Case–control	Bipolar	8	42.00	9.90	3	5	–	–	19.00	9.00	Total weighted Waldrop scale	2.90	2.000				
			Control	14	39.60	17.70	7	7	–	–				2.90	1.900				

*Cn, country; S.T., study type; S.S., sample size*.

### Assessment of MPAs

In the reviewed studies, the Waldrop scale (30) and the Méhes Scale (7, 31, 32) were the applied measures for the evaluation of MPAs were. The Méhes Scale includes 57 minor signs (7, 8, 19). The original Waldrop scale contains 18 minor physical anomalies (30) from the following regions: head, eyes, ears, mouth, hands, and feet. The abnormalities are scored as present (1) or absent (0). The variables—fine electric hair, head circumference, epicanthus, intercanthal distance, low seated ears, and high or arched palate—are scored by 1 or 2, according to severity.

Akabaliev et al. (27) used a changed version of the Waldrop scale containing 19 items. Separate items were defined, such as adherent ear lobes and lower edges of the ears that extend backward/upward due to the increased prevalence of the former and the occasional occurrence of the latter. They graded randomly furrowed tongue by 1 (a normal variant) and transversely furrowed tongue (frequently observed in pathological conditions) by 2. Intercanthal distance abnormality was also scored in cases of hypotelorism. The intercanthal distance and the head circumference were scored 1 if it differed from the healthy controls by 1.5–2 standard deviations and 2 if they differed by more than 2 standard deviations.

Green et al. (28) used another modified version of the Waldrop scale. They modified the scale for head circumference and intercanthal distance by scoring one point if the measurements differed by more than 1.5 standard deviations from the normal controls. The original scale was more liberal: one point was given if 1 standard deviation was detected and two points if 2 standard deviations were detected.

Alexander et al. (29) calculated the total weighted Waldrop score, in which for items 3 (head circumstances) and 5 (intercanthal distance) subjects were given a weighted score of 1 for measurements 1 standard deviation above or below the mean values reported for Caucasians and a weighted score of 2 above or below these means.

Berecz et al. (26) used the Méhes Scale for the evaluation of MPAs. As the Méhes Scale includes 57 minor signs, for the subsequent meta-analysis we took into consideration those 18 items which overlap in the two scales.

### Data Analysis and Grade Approach

Standardized mean differences (SMD) were calculated with the 95% confidence interval (CI) between the two groups for total MPA (MPA-T), Craniofacial MPA (MPA-CF), and Periphery MPA (MPA-P) scores because there was no single-score system. Pooled estimates were calculated with the random-effect model by using the DerSimonian–Laird method (1). Results of the meta-analysis were displayed graphically using Forest plots. Heterogeneity was tested by using Cochrane's Q and the *I*^2^ statistics, where *I*^2^ = 100% × (Q–df)/Q and represents the magnitude of the heterogeneity (moderate: 30–60%, substantial: 50–90%, considerable: 75–100%) (2). All meta-analytical calculations were performed by Stata 15 data analysis and statistical software (Stata Corp LLC, College Station, TX, USA).

We present the overall quality of the evidence using the GRADE approach as recommended by the Cochrane Handbook for Systematic Reviews of Interventions (33). The quality of the evidence was based on four factors: (1) limitations of the study design or the potential for bias across all studies that measure that particular outcome, (2) consistency of results, (3) directness (generalizability), and (4) precision (sufficient data). The quality of evidence was downgraded by one level if one of the factors described above was not met. Likewise, if two or three factors were not met, we downgraded the level of evidence by two or three levels, respectively. Thus, the GRADE approach resulted in four levels of quality of evidence: high, moderate, low, and very low.

## Results

**Figure 1:**
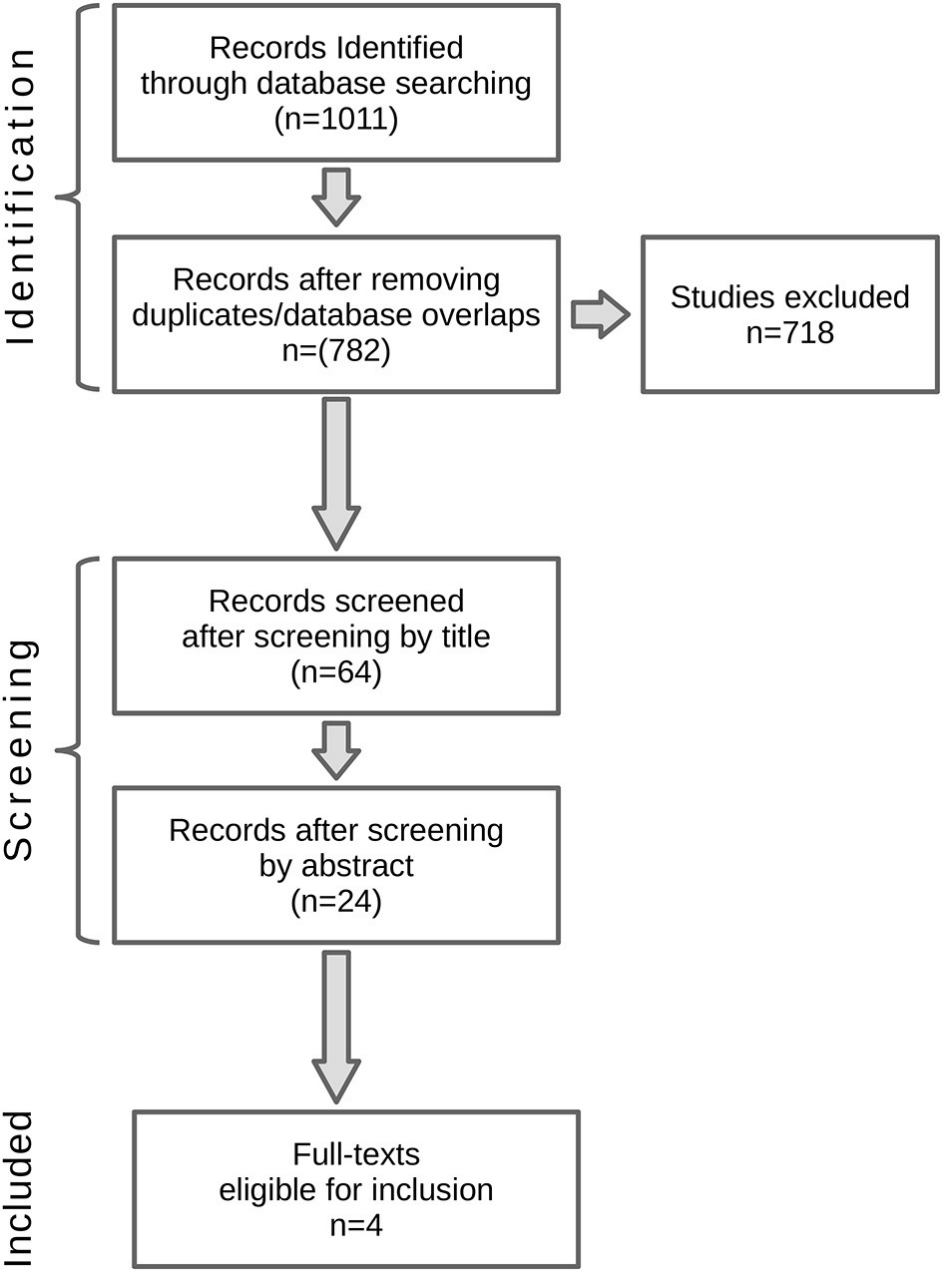
Flowchart of the study selection process.

The initial search strategy yielded 1,011 studies. Numbers of records in each database are as follows: Pubmed: 195, Cochrane: 35, Embase: 353, Web of Science: 428. After the filtration of the duplicates, 782 publications were checked for eligibility criteria. After checking the publications by title, 64 studies passed the eligibility criteria. After checking the publications by abstract, 24 of them passed eligibility criteria. Figure 1 presents the flowchart of the publication selection process.

Four studies involving 155 patients with BPD and 187 HC passed the inclusion criteria (Table 2). For the investigation of MPAs in the head and facial regions, two studies involving 121 BPD patients and 133 HC could be included.

**Figure 2:**
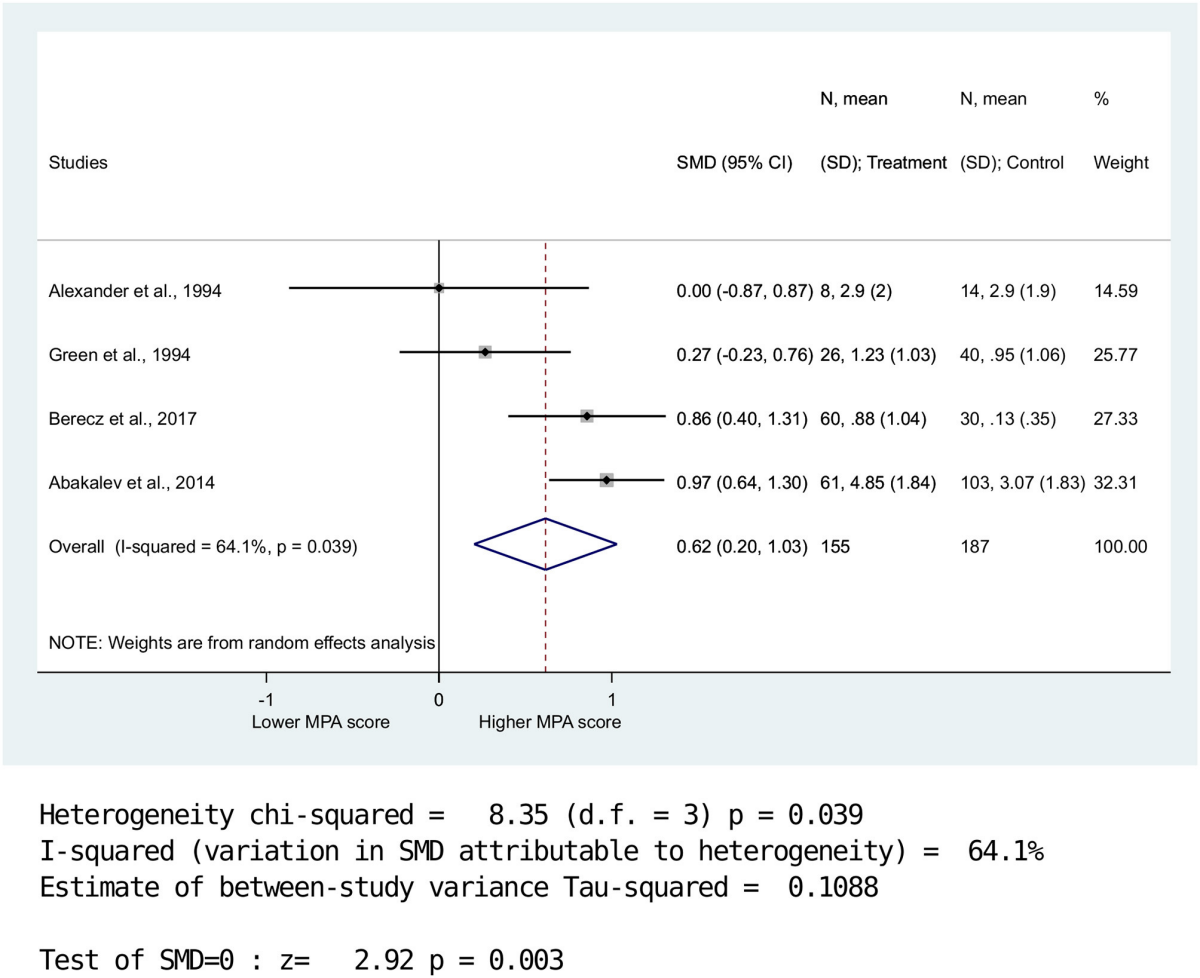
Standardized mean difference of minor physical anomalies in patients with bipolar disorders vs. healthy controls. There was significantly higher total MPA scores in BPD compared to HC. (SMD = 0.62, 958%; CI: 0.20-1.03, p = 0.003).

**Figure 3:**
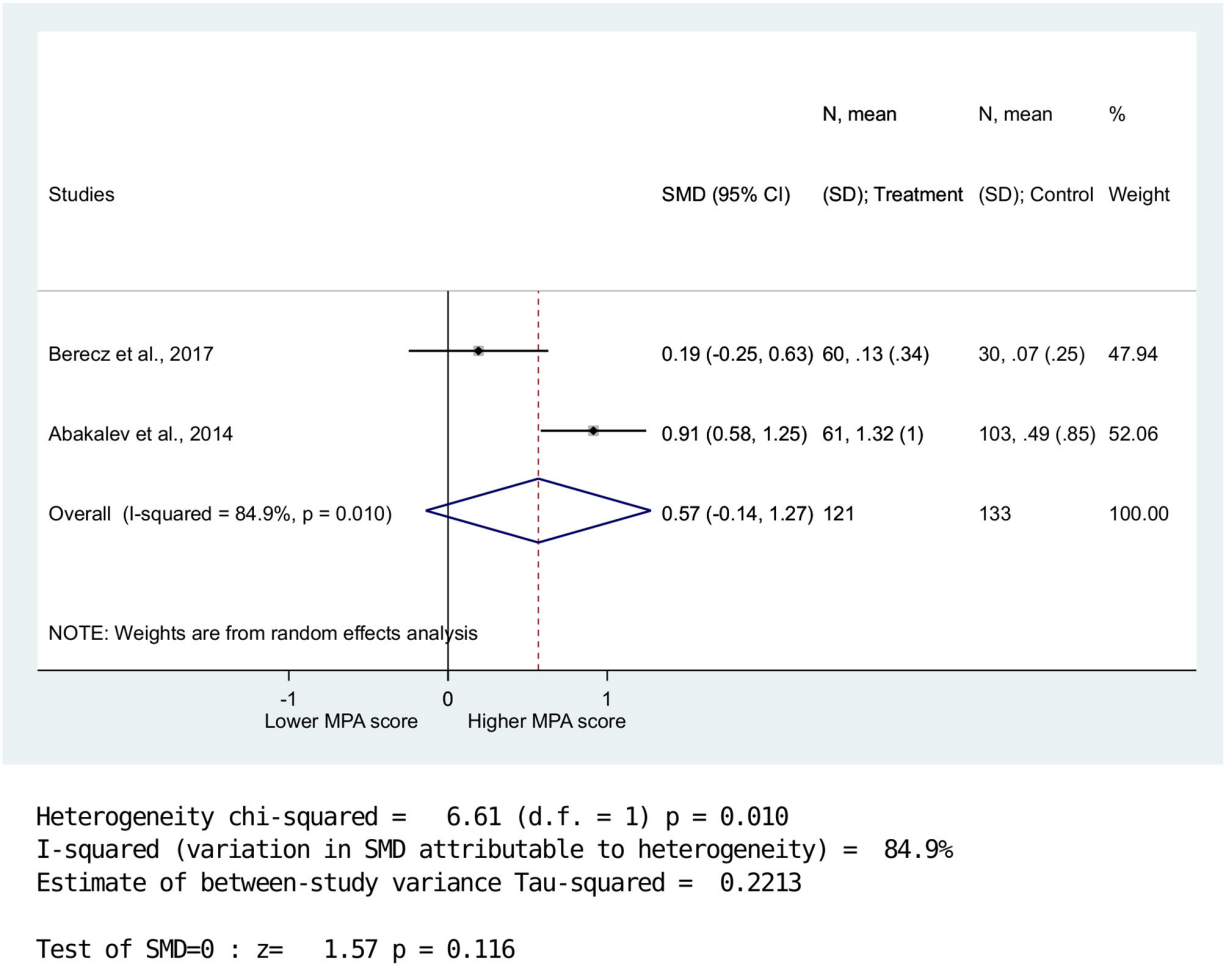
Standardized mean difference of minor physical anomalies in the peripheral regions in patients with bipolar disorders vs. healthy controls. There was no significant between-group difference between BPD and HC in MPA-P scores. (SMD = 0.57,95%; CI: −0.14-1.27, p = 0.116).

**Figure 4:**
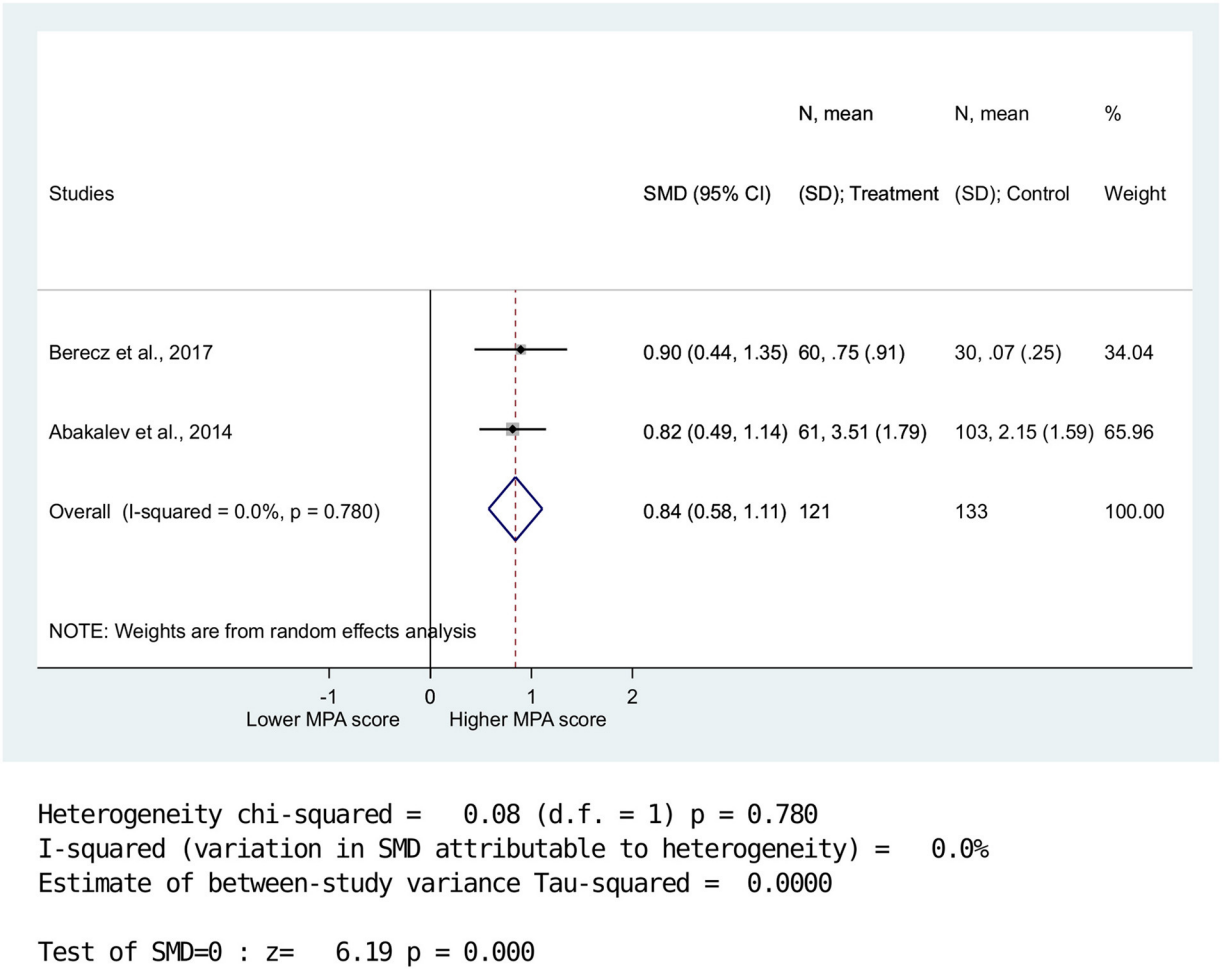
Standardized mean difference of minor physical anomalies in the head and facial regions in patients with bipolar disorders vs. healthy controls. There was significantly higher MPA-CF score in BPD compared to CG. (SMD = 0.84,958%; CI:0.58-1.11, p < 0.001).

Overall MPA scores (*n* = 4) were significantly higher in the BPD group compared with the HC group (SMD = 0.62, 958% CI: 0.20–1.03, *p* = 0.003) (Figure 2). Data were further analyzed for MPA-P and MPA-CF (*n* = 2). Results showed no significant between-group differences for MPA-P (SMD = 0.57, 95% CI: −0.14–1.27, *p* = 0.116) (Figure 3). Nevertheless, there was a significant difference between BPD patients and HCs for MPA-CF (SMD = 0.84, 958% CI: 0.58–1.11, *p* < 0.001) (Figure 4).

High heterogeneity was detected for MPA-P (Q = 6.61; DF = 1; *I*^2^ = 64.1%; *P* < 0.01), whereas lower heterogeneity was observed for overall MPA (Q = 8.35; DF = 3; *I*^2^ = 84.9%; *P* < 0.039) and MPA-CF (Q = 0.08; DF = 1; *I*^2^ = 0.0%; *P* < 0.78).

We made a visual assessment of the funnel plot (Figure 2) to evaluate publication bias, because we included four studies in our meta-analyses (33).

There was a low risk of bias in items 1, 7, and 8 (item 1: is the case definition adequate?, item 7: ascertainment of exposure, item 8: same method of ascertainment for case and controls); it received 100% (Table 3). With regard to representativeness of the case, 50% of the articles represented a low risk of bias, while 50% had an unclear risk of bias. With regard to a comparison of age, 100% of the articles presented significant difference in the ages between the groups. With regard to a comparability of sex, 50% of the articles presented significant difference in the ages between the groups. Only 75% of the studies presented low risk of bias in item 4 (definition of controls).

**Table 3:**
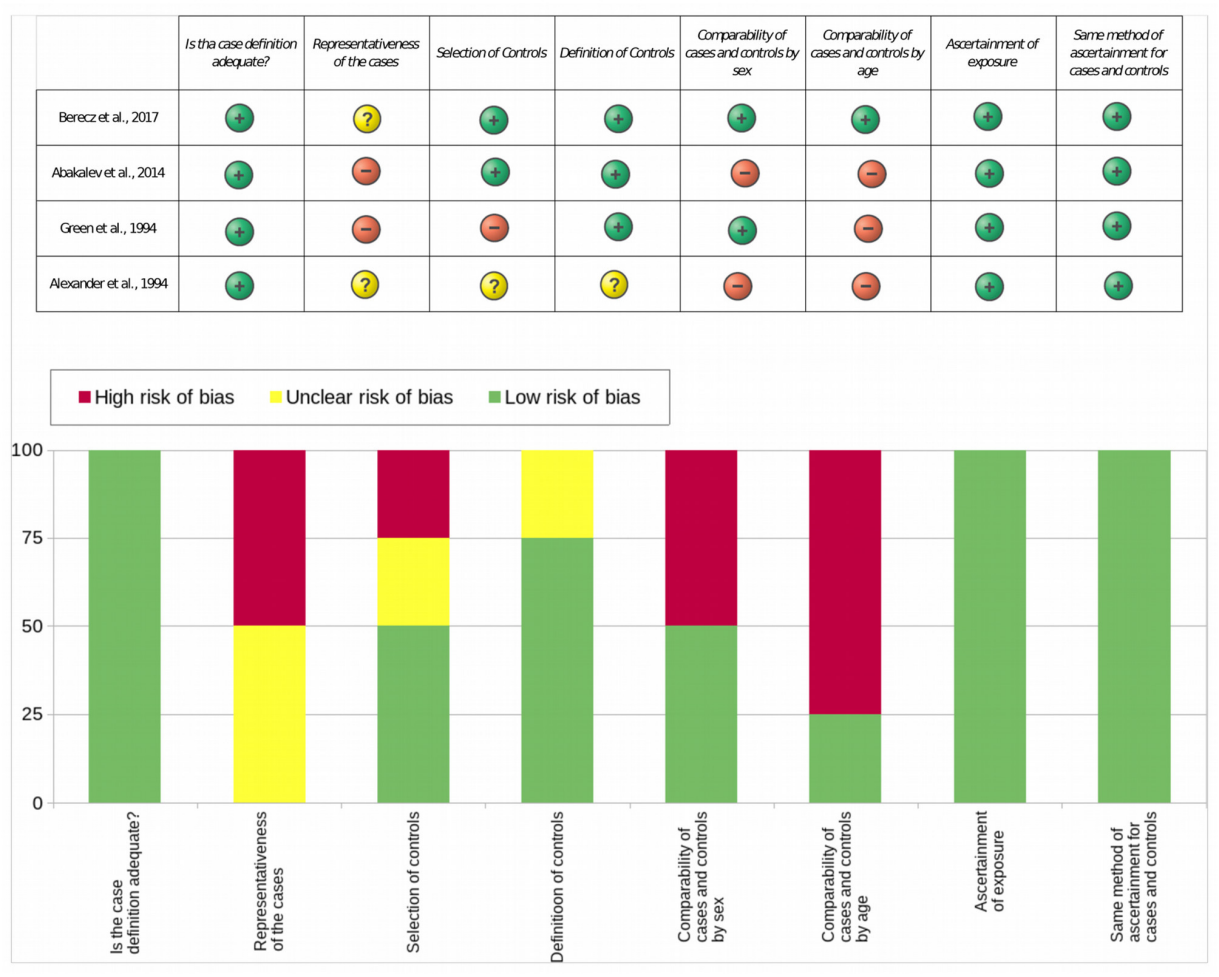
Results of the assessment of the quality of included studies by NOS.

Overall judgment of the quality of the evidence using the GRADE approach is summarized in Table 4. We found low/very low quality of evidence of the results.

**Table 4:** Overall judgment of quality of evidence using the GRAD approach.

**Outcome measure**	**N of studies**	**Limitations**	**Inconsistency**	**Indirectness**	**Imprecision**	**Quality of evidence**
Number of total MPAs in BPD compared to HC	4	Serious	No serious inconsistency	No serious indirectness	Serious	Low
Number of MPA-CF in BPD compared to HC	2	Serious	No serious inconsistency	No serious indirectness	Serious	Very low
Number of MPA-P in BPD compared to HC	2	Serious	No serious inconsistency	No serious indirectness	Serious	Very low

## Discussion

Our meta-analysis showed that the MPA scores of patients with BPD were significantly higher compared to HC. BPD patients' MPA-P scores did not significantly differ from those of healthy controls. In contrast, BPD patients' MPA-CF scores were significantly higher compared to HC subjects. These results support our hypothesis that MPAs are more common in patients with bipolar affective disorders than in healthy subjects and the higher rate of MPAs are found predominantly in the head and facial regions. Based on earlier findings, we suggest that the disease of those patients who have higher numbers of MPAs is due to impaired brain development, resulting in worse disease prognosis (26).

Evidence indicates that schizophrenia and BPD share certain genetic and pathophysiological similarities (4, 34). These similarities led to the assumption that similar neurodevelopment abnormalities might play a role in the pathophysiology of BPD. Increasing the risk of BPD, various genetic and environmental factors can lead to structural and functional brain changes during the prenatal period. The hypothesis for aberrant neurodevelopment in BPD is suggested by results found in different studies (35). Obstetric complications (36), prenatal influenza infection (37), prenatal famine (38), and urban (39) and winter (40) birth precede the onset of BPD. Lower IQ (41), delayed attainment of developmental milestones (42), emotional problems, and interpersonal difficulties (43) have also been implicated as premorbid neurobehavioral precursors. There are structural neuroimaging studies (44, 45) also investigating the neurodevelopmental theory of the etiology of BPD.

Brain development happens in a hierarchical and sequential way, and there is a critical period of vulnerability to teratogens of each organ that may result in developmental disorder. There is a possibility that traumas, which occur at specific periods during gestation, increase the risks for atypical brain development and functional psychoses. The identification of MPAs that are related to specific behavior disorders should help to identify which part of the brain may be affected and thus may be involved in a given psychiatric disorder. This identification would be achieved by establishing the correspondence between the period of vulnerability for the brain developmental phases and the affected organ.

Advances in the identification of the critical periods for insults to the brain can be expected by the use of more detailed MPA scales with clear distinction between minor malformations and phenogenetic variants. A clear differentiation between morphogenetic events developing during and after organogenesis is required.

As our research group (7, 9, 10, 19, 29, 46) and other researchers (47) have reported in previous studies, contradictions between studies on MPAs among patients with BPD might be related to the difficulties in the use of the Waldrop scale for the detection of MPAs. It has poor internal consistency due to the heterogeneity of the anomalies in terms of location, character, and time of prenatal development (47). It contains 18 MPAs (30) while the literature lists more than 50 of them (31, 32). Another problem with the scale is that it does not make any differentiation between minor malformations and phenogenetic variants (31, 32, 46). Minor malformations are qualitative deficits of the embryogenesis, which arise during organogenesis. They are developmental field deficits and usually all-or-none anomalies. In contrast, phenogenetic variants are quantitative defects of the morphogenesis and appear after organogenesis.

We conclude that the findings of the present meta-analysis suggest an early insult during brain development in BPD. It might be possible that the early abnormal development represented by MPAs reflects an early developmental pathway toward later mental impairments. Moreover, the more frequent appearance of MPA-CF scores supports the theory that anomalies of the head and the mouth may have more relevance to a hypothetical neurodevelopmental impairment, which suggests a worse prognosis of the disease. However, according to the low/very low quality of evidence, results of the present meta-analyses should be interpreted with caution.

Our meta-analysis has some limitations as well. The greatest limitation is the low number of eligible publications included, which caused higher heterogeneity. Several studies that investigated MPAs in bipolar disorders do not provide sufficient data for meta-analyses, so for the meta-analyses of MPA-P and MPA-CF only two studies (involving 121 patients with bipolar disorders and 133 healthy controls) could be included. However, the Cochrane Handbook for Systematic Reviews states that meta-analysis is the statistical combination of results from two or more separate studies (48); moreover, Valentine et al. also stated that at least two studies are eligible for a meta-analysis, because all the other synthesis techniques are less transparent and are less likely to be valid (49).

We believe that in order to have more consistent evidence about MPAs in BPD, further investigations are needed. In addition, minor malformations and morphogenetic variants should be differentiated, and we recommend the use of the more detailed Méhes Scale for the identification of MPAs in further studies.

## Data Availability Statement

The original contributions presented in the study are included in the article/supplementary material, further inquiries can be directed to the corresponding author/s.

## Author Contributions

EV and AH: data collection, data analysis, writing article, and manuscript revision. AS: data analysis, writing article, and manuscript revision. PH, DK, NF, JS, and AM: data analysis, writing article, and manuscript revision. TT and RH: writing article and manuscript revision. All authors contributed to the article and approved the submitted version.

## Conflict of Interest

The authors declare that the research was conducted in the absence of any commercial or financial relationships that could be construed as a potential conflict of interest.
